# Effects of Dietary Supplementation of *Spirulina platensis* on the Immune System, Intestinal Bacterial Microbiome and Skin Traits of Mink

**DOI:** 10.3390/ani13020190

**Published:** 2023-01-04

**Authors:** Anna Maria Iatrou, Sofia Michailidou, Georgios A. Papadopoulos, Hara Afaloniati, Maria K. Lagou, Maria Kiritsi, Anagnostis Argiriou, Katerina Angelopoulou, Theofilos Poutahidis, Paschalis Fortomaris

**Affiliations:** 1 Laboratory of Animal Husbandry, School of Veterinary Medicine, Faculty of Health Sciences, Aristotle University of Thessaloniki, 54124 Thessaloniki, Greece; 2 Center for Research and Technology, Hellas Institute of Applied Biosciences, Thermi, 57001 Thessaloniki, Greece; 3 Laboratory of Biochemistry and Toxicology, School of Veterinary Medicine, Faculty of Health Sciences, Aristotle University of Thessaloniki, 54124 Thessaloniki, Greece; 4 Laboratory of Pathology, School of Veterinary Medicine, Faculty of Health Sciences, Aristotle University of Thessaloniki, 54124 Thessaloniki, Greece; 5 Department of Food Science and Nutrition, University of the Aegean, 81400 Lemnos, Greece

**Keywords:** Spirulina, mink, gut, microbiome, immune system, skin quality

## Abstract

**Simple Summary:**

Many dietary practices have been previously applied in mink farming to improve the health and productivity of the animals. *Spirulina platensis* (Spirulina), a natural feed supplement, has demonstrated beneficial effects on the health and productivity of various animal species. To the best of our knowledge, the effect of Spirulina supplementation on the health of mink has not been previously studied. To address this dearth of knowledge, we investigated the effects of Spirulina supplementation on the immune system, gut bacterial microbiome and skin of mink. Spirulina decreased the markers of subclinical inflammation of the mink gastrointestinal system. Moreover, differences in the bacterial communities’ compositions among groups were observed. Dietary inclusion of Spirulina at a dose of 100 mg/kg of body weight did not affect either the growth of animals or the skin and pelt quality. Overall, it can be suggested that Spirulina can be used with mink to promote their intestinal health and immune status. However, further studies may be required to investigate the effect of other levels of Spirulina dietary supplementation.

**Abstract:**

The impact of dietary inclusion of *Spirulina platensis* on the immune system, intestinal microbiome and skin of mink was investigated. Forty-eight animals were equally separated into four groups. Groups B and D were control animals, while groups A and C had their feed supplemented daily with 100 mg/kg of body weight Spirulina. Mink in groups A and B were descended from dams supplemented with spirulina during their reproductive period, while those in groups C and D were descended from dams fed the control diets. Fur growth rate and quality were graded semi-quantitatively. Fecal microbiome analysis, skin thickness histomorphometry, immunohistochemical labeling and counts of immune cells in the colon, mesenteric lymph nodes and spleen and quantitative gene expression analysis of cytokines in the colon were performed. Skin thickness, fur growth rate and skin quality were similar among groups (*p* > 0.05). However, differences were observed among groups concerning the relative and differential abundance of bacterial species. Tgf-β expression was lower in group A, whereas IL-β1 was lower in group C compared to group B (*p* < 0.05). Group D had significantly lower numbers of inflammatory cells in the colon and mesenteric lymph nodes. The results revealed that Spirulina decreased indices of subclinical inflammation in mink gut, while differences in the bacterial communities among groups were observed.

## 1. Introduction

Nutrition affects gut microbiota, which, in turn, affect overall host function and, especially, the metabolic and immunologic responses of mammalians [1,2,3]. Gut bacterial composition is basically shaped by environmental and nutritional factors, as well as by living conditions and host status [4]. Alterations in the balance of gut microbiota can predispose animals to the development of diseases and various pathological conditions [5]. 

Recent evidence published elsewhere about the function of the physiological axis between the gastrointestinal system and the brain (the “gut–brain axis”) suggests ongoing interaction between the functions of the gastrointestinal tract, its microbiome and the brain [6,7,8]. Several gut bacteria have also been shown to interact with the immune system and affect remote organs, including skin. According to recent studies on mice, the effects of orally administrated bacteria result in systematic responses and extend to organs and tissues beyond the gastrointestinal system [9,10,11]. Recent research revealed the physiological interactions and signaling between the gut and its microbiota, as well as the brain and skin, including its components, such as sebaceous glands and hair follicles [6,12]. Older studies demonstrated that gut bacterial communities can positively affect the response to disturbed skin-barrier function and contribute to skin allostasis [13]. Elsewhere, it was found that probiotics may affect skin health through their effect on inflammatory cells, as well as cytokines [14]. 

The gastrointestinal tract in mink is simple and short compared to other mammalian species, but the caecum is missing [15]. Feed transit time is subsequently fast, which makes microflora in the intestinal tract unstable and microbial activity in the large intestine low [1,16,17,18,19]. It is believed that the role of gut microbiota in carnivores is limited and mainly characterized by enzymatic digestion [20]. Knowledge of the composition of the normal bacterial community in the intestinal tract of mink is important for both understanding the enteritis outbreaks in animals and for finding suitable probiotics or feed additives for use in mink farming [1,19]. Older conventional culture-based studies on mink revealed that the colon consists of equal counts of aerobic and anaerobic bacterial species [21]. 

*Spirulina platensis* (Spirulina) is a cyanobacterium widely used as a nutritional supplement due to its antibacterial, antioxidant, antivirus, anti-inflammatory and immunomodulation activity [22,23,24,25]. Spirulina is rich in proteins, vitamins, essential amino acids and fatty acids [26]. The finding that supplementation with Spirulina affects the gastrointestinal microbiota of mice is of particular interest [2,4]. Elsewhere, Spirulina supplementation in dogs was found to increase the stability of microflora in the gut and enhance immune response and gut health [27]. Spirulina supplementation in mice inhibited overproduction of pro-inflammatory cytokines, enhanced antioxidant activity in the colonic mucosal barrier and prevented ulcerative colitis [28]. The antioxidant activity and high protein content of Spirulina play major roles in wound healing in the skin by scavenging reactive oxygen species (ROS) and promoting the expression of collagen in granulation tissue, respectively [29]. 

To date, there is limited evidence regarding the effects of dietary supplements in mink on gastrointestinal function and health. Based on the properties of Spirulina, it could be hypothesized that use as a dietary supplement could affect minks’ overall health condition and their productive characteristics, such as pelt quality, through alterations to the gut microflora. To further investigate whether these parameters for growing mink could be affected by the diet fed to dams during the reproductive period, the long-term effects of dietary supplementation with Spirulina were also investigated. For this reason, the offspring mink used in the present study originated from dams fed diets that were either supplemented with Spirulina or not. The present study investigated the effects of dietary supplementation with Spirulina on growing mink and the alterations to their intestinal microbiota, immune system, growth rate, skin and pelt quality. 

## 2. Materials and Methods

### 2.1. Animals

Animals used in this research were raised under standard mink-farming conditions. The study was carried out in a commercial mink farm in northern Greece. In total, 300 healthy brown mink (Mustela vison) free of Aleutian disease virus were used. Animals were kept under conventional mink-farm conditions and fed a standard mink diet. Mink were housed in roofed sheds with open sides in individual standard mink cages (30 cm width × 92 cm length × 46 cm height) bedded with straw with attached wooden nest boxes. The experimental period lasted ten months (1st February to end of November).

### 2.2. Animal Design, Diet and Sample Collection

**Figure 1:**
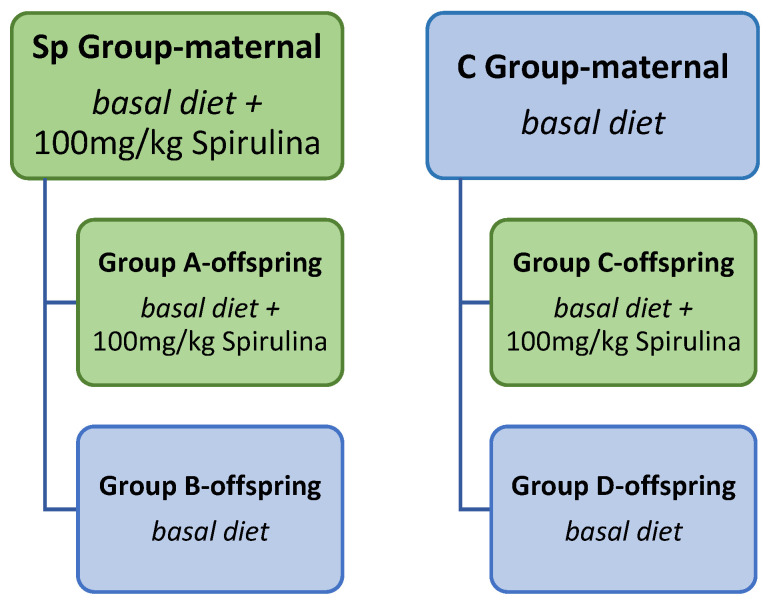
Experimental layout.

The study involved dietary supplementation with Spirulina in the diets of reproductive and growing mink. The experimental layout is shown in Figure 1.

Firstly, 100 female animals were randomly and equally allocated to a control group (Con group, *n* = 50) in which minks were fed the basal diet and a Spirulina group (Sp group, *n* = 50) in which the basal diet was supplemented with 100 mg of Spirulina (powder) per kg of body weight (BW). Inclusion of Spirulina started 1 month prior to the mating season (early February) and lasted until the weaning of kits in late June. Doses were calculated monthly according to the animals’ average body weight. Kits started to have access to solid feed in addition to milk at four weeks postpartum and were fed the same feed as their dams until weaning. After the weaning period, one female and one male mink were housed in growth cages until their pelting, whereas dams were removed from the experiment.

Next, after weaning, 200 offspring (100 females, 100 males) from the Sp and Con groups were equally allocated to four groups. The first two groups (A and B) descended from the Sp group. Group A (*n* = 50, 25 females, 25 males) received daily supplementation with 100 mg/kg of BW Spirulina until the pelting period, while group B (*n* = 50, 25 females, 25 males) consisted of mink that were fed the basal diet without supplementation with Spirulina (Figure 1). 

Groups C and D descended from the experimental group of control dams (Con group). Group C (*n* = 50, 25 females, 25 males) consisted of animals that received 100 mg/kg of BW Spirulina daily with their feed until the pelting period, while group D (*n* = 50, 25 females, 25 males) consisted of animals that did not receive Spirulina.

The nutrient analysis of feed samples was carried out with four samples of control feed. The samples were collected directly from the feed silos before distribution to the feeding machine. The feed nutrient chemical analysis was performed at the Laboratory of Animal Nutrition at the School of Veterinary Medicine, Faculty of Health Sciences, of Aristotle University with a near-infrared reflectance (NIR) DA7250 Perten Instrument (Perten, Instrumentvägen 31, SE-126 53 Hägersten, Sweden). According to the analysis, moisture found in the feed amounted to 3.41 g/kg, protein to 35.70 g/kg, fat to 21.74 g/kg and ash to 5.28 g/kg. The Spirulina were supplied by Hellenic Spirulina Net, and Spirulina powder was first dissolved in 500 mL of fresh water and then mixed with the basal feed for mink. Animals had free access to drinking water from a nipple drinker throughout the experimental period. The total experimental period of the second experimental phase was 5 months.

### 2.3. Body Weight

To study the effect of Spirulina on the growth of mink, the body weights of 200 animals (25 females and 25 males from each of the four groups) were measured once per month from kit weaning to pelting. Five measurements were conducted. Body weight differences between consecutive measurements were calculated. 

### 2.4. Hair Re-Growth Assessment

For the assessment of hair re-growth, ten mink (five females and five males) from groups C and D, respectively, were used. A square area measuring 5 × 5 cm in the caudal ventral back area was shaved using a normal electric clipper. The shaved area was photographed every 3 days until the 27th day post-shaving. Images were used by two expert fur quality evaluators for the subjective evaluation and fur re-growth was scored on a 0–5 scale at each time point.

### 2.5. Grading

Live grading of skin quality was performed in the farm by skilled professional pelt graders on a scale from 1 to 5 (1: lowest quality, 5: best quality) according to the following criteria: density of underfur, body length, pelt nap length, overall impression of the fur.

### 2.6. Euthanasia and Necropsy

In November, during pelting season, 48 mink (6 females and 6 six males from each of the four groups) were killed with carbon monoxide gas in accordance with European standards and a full necropsy was performed. 

### 2.7. Fur Quality Scoring

The skins of the animals were collected and sent to Saga Furs Auction House in Finland, where they were graded and sold in an auction. The size of the pelt, size of the nap, overall quality of the skins and price were recorded. Categorization and scoring of skins were undertaken according to Saga Furs standards. 

### 2.8. Histopathology, Immunohistochemistry and Morphometry

For histopathological and immunohistochemical analyses, skin, colon, mesenteric lymph node (MLN) and spleen samples were collected from standardized anatomical sites. Tissues were fixed in 10% neutral-buffered formalin, embedded in paraffin, sectioned at 4–5 μm (SLEE Medical GmbH, Cut 5062, Mainz, Germany) and stained with hematoxylin and eosin or subjected to immunohistochemical labeling (IHC). 

Primary antibodies for IHC labeling of immune cells in the colon, MLN and spleen tissue sections included rabbit antibodies against myeloperoxidase (MPO, ThermoFisher Scientific/Lab Vision, Fremont, CA, USA), PAX-5 (Abcam, Cambridge, UK) and CD3 (Cell Marque, Rocklin, CA, USA). Heat-induced antigen retrieval was performed with citrate buffer, pH 6, for myeloperoxidase (MPO); EDTA buffer, pH 8, for PAX5; and with CC1 epitope retrieval solution (Ventana Medical Systems, Inc., Oro Valley, AZ, USA) for CD3. Rabbit primary antibody binding was detected with goat anti-rabbit polymer HRP (ZytoChem Plus, Berlin, Germany). Color was developed with diaminobenzidine substrate-chromogen (Biogenex, Fremont, CA, USA) and tissue sections were counterstained with hematoxylin.

Skin sections from 12 randomly selected animals (6 male and 6 female) were used for histomorphometrical measurements of skin thickness. Colon, MLN and spleen sections from six randomly selected animals (three male and three female) were used for the quantitative assessment of IHC-stained immune cells. Multiple low-magnification (×2) images of skin containing longitudinally oriented hair follicles or high-magnification (×20) images of the colonic lamina propria, MLN paracortex and red pulp and non-follicular areas of the spleen were captured, and four (skin thickness) or three (cell counts) images were randomly selected from each animal’s tissue section for measurements. The measurement of the distance involving the epidermis, dermis and subcutaneous tissue and the counting of IHC-positive cells in each image was undertaken with the Image J image processing and analysis program (NIH, Bethesda, MD, USA). Forty-eight distance and eighteen cell-count values were recorded from each experimental group for statistical analyses.

### 2.9. Quantitative Gene Expression Analysis

Total RNA was extracted from tissue samples using the NucleoSpin Total RNA Isolation kit (Macherey-Nagel, Duren, Germany) according to the manufacturer’s instructions. After spectrophotometric determination of RNA concentration and quality, samples were stored at −80 °C until use. Reverse transcription was carried out using the FastGene Scriptase II cDNA kit (Nippon Genetics, Tokyo, Japan). Five hundred nanograms of total RNA was used as starting material for cDNA synthesis. Real-time PCR based on the SYBR Green chemistry was used to quantitatively analyze the expression of TGF-β, IL1-β, IFN-γ, IL-6 and TNF-α. The housekeeping gene glyceraldehyde-3-phosphate dehydrogenase (GAPDH) was used as an internal control. Primers were designed using Primer3Plus (version 3.2.6) according to nucleotide sequences available in GenBank. Primer sequences, their positions within the corresponding genes, GenBank accession numbers and amplicon sizes are presented in Table 1. PCR amplification was performed in 10 μL reaction mixtures containing 2 μL cDNA, 1× KAPA SYBR FAST qPCR master mix (KAPA BIOSYSTEMS, Woburn, MA, USA) and 100–300 nM of each primer pair (Table 1). The temperature cycling on a PCRmax Eco 48 real-time PCR system (PCR max, Staffordshire, UK) included 35–40 cycles, consisting of denaturation at 95 °C for 10 s and annealing/extension at temperatures ranging from 61 to 62 °C for 20 s (Table 1). Each PCR reaction was initiated with 3 min denaturation at 95 °C and terminated with sequential readings between 55 and 95 °C (increment of 0.3 °C) in order to generate the melting curve and verify amplicon specificity. For relative quantification of gene expression, we used the comparative Ct method [30]. 

### 2.10. Stool Bacterial Flora Microbiome Analysis

#### 2.10.1. Library Construction and Sequencing

DNA was extracted from 24 samples using the NucleoSpin DNA Stool (Macherey-Nagel, Duren, Germany) kit. Library construction and sequencing were performed by MR DNA (www.mrdnalab.com, Shallowater, TX, USA). In brief, libraries were constructed by amplifying the V4 hypervariable region of the 16S rRNA gene using primers 515 and 806 [31]. Prior to sequencing, libraries were multiplexed using unique dual indices and pooled together in equal proportions based on their molecular weight and DNA concentrations. Sequencing was performed on a MiSeq platform using the MiSeq^®^ reagent kit v3 (2 × 300 cycles) following the manufacturer’s guidelines. 

#### 2.10.2. Bioinformatics

Bacterial communities in the gut microbiome were analyzed using Qiime2 v-2020.2 (Quantitative Insights into Microbial Ecology 2, https://qiime2.org/) [32]. The DADA2 pipeline implemented in Qiime2 was applied to process raw sequences and obtain representative amplicon sequence variants (ASVs); raw reads were trimmed, quality filtered, merged, dereplicated and denoised into unique sequences [33]. Chimeric sequences were also identified and removed from the dataset. The SILVA (138) database was used for bacterial ASV taxonomic classification. ASVs classified as Archaea, chloroplasts or mitochondria and those with unidentified annotation were excluded from downstream analysis. The ASV table was imported into R programming language v4.1.15 for further processing and visualization of the results using the phyloseq R package [34,35]. Heatmaps were used to depict ASV frequencies normalized to 100% as abundance estimations within each sample and visualized by combining functions provided by the ggplot2 R package [36]. Identification of differentially abundant (DA) taxa among the different diet groups versus the control group was performed with DESeq2 v1.34.0 [37] in R. Size factors were calculated using the type “poscounts” and data were normalized to the log2fold change. Subsequently, log fold-shrinkage method was performed with the “apeglm” to adjust for low counts and highly dispersed features [38]. Those features with an adjusted *p* value < 0.01 were deemed significant and further evaluated.

### 2.11. Statistical Analyses

Morphometric counts and hair re-growth assessment analysis were compared between experimental groups with Mann–Whitney U analysis. Statistical analyses of skin quality, grading from live animals and sizes and naps of pelts were conducted using a Chi-squared independence test. For the values for body weight and skin price, a univariate analysis of variance (ANOVA) with treatment group as a fixed factor was performed. Weight differences between mink during the experimental period were analyzed with an unpaired *t*-test. Statistical significance was set at *p* < 0.05, whereas values between 0.05 < *p* < 0.10 were considered as a “trend”. For the analyses of the hair re-growth assessment, grading from live animals, skin quality, sizes and naps of pelts, skin price and body weight, the software SPSS v. 25.0 (IBM Corp., Chicago, IL, USA) was used. All the rest of the analyses were performed with Graphpad Prism (version 9.1.2 for Windows^®^, GraphPad Software, San Diego, CA, USA). Data representation was undertaken with bar graphs depicting the mean and standard error of the parameter assessed for each experimental group.

## 3. Results

### 3.1. Body Weight

**Figure 2:**
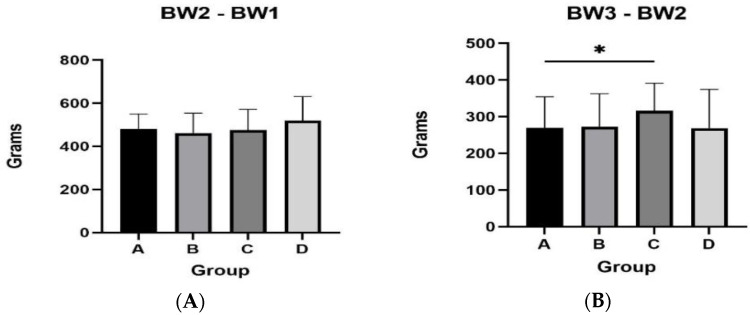

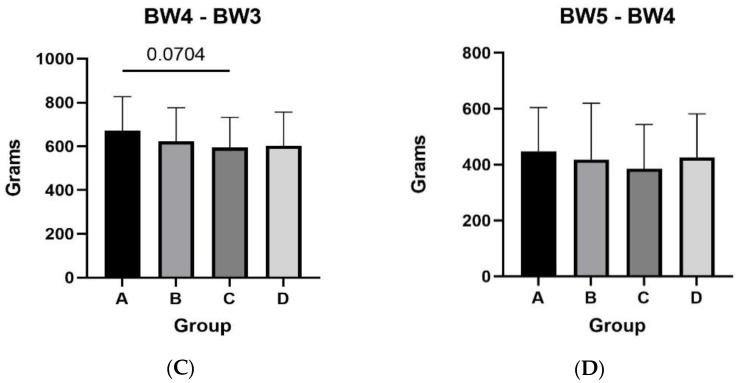
Body weight differences for female mink from the four experimental groups for the following intervals: at second month of the experimental period and first month (panel **A**); at third month of the experimental period and second month (panel **B**); at fourth month of the experimental period and third month (panel **C**); at fifth month of the experimental period and fourth month (panel **D**). * *p* < 0.05.

The female mink from group A presented a tendency for higher body weight during the second measurement (*p* = 0.077) (Table 2). During August (BW3 and BW2), the body weight gain in group C was higher compared to group A (*p* = 0.046), and there was a tendency for higher body weight gain compared to groups Β and D (A vs. B, *p* = 0.071; A vs. D, *p* = 0.075) (Figure 2). During the last month of supplementation with Spirulina (BW5 and BW4), group D presented a tendency for higher body weight gain in comparison to animals from groups A and B (D vs. B, *p*= 0.071; D vs. B, *p* = 0.058). However, no statistically significant difference in the final body weight for the four groups (*p* > 0.05) was presented. 

**Figure 3:**
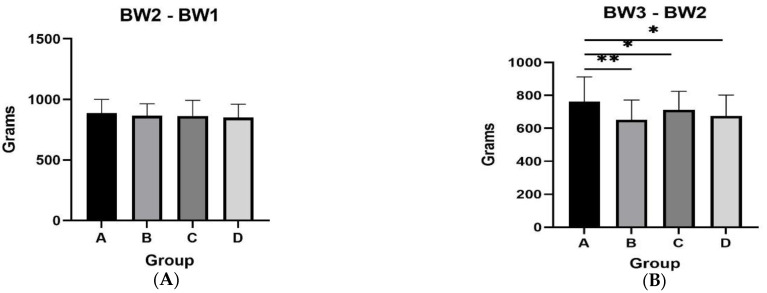

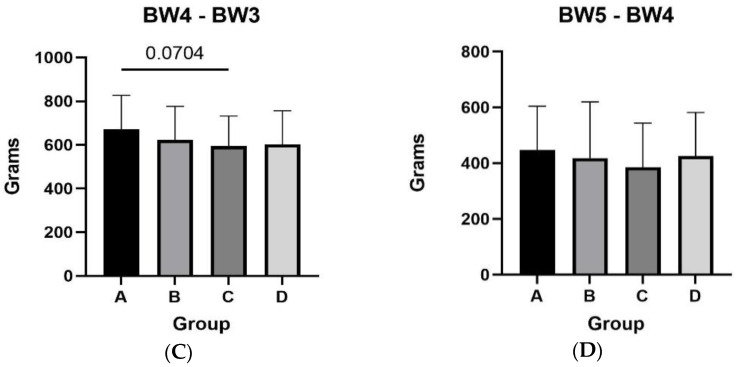
Body weight differences for male mink. Weight differences for male mink from the four experimental groups for the following intervals: at second month of the experimental period and at first month (panel **A**); at third month of the experimental period and at second month (panel **B**); at fourth month of the experimental period and at third month (panel **C**); at fifth month of the experimental period and at fourth month (panel **D**).* *p* ≤ 0.05; **: *p* < 0.01.

Regarding male mink, during August (BW3 and BW2), group A presented higher body weight gain compared to the rest of the groups (A vs. B, *p* = 0.006; A vs. C, *p* = 0.033; A vs. D, *p* = 0.058) (Figure 3). The same group also presented a tendency for higher body weight gain in September (BW4 and BW3) compared to group C (*p* = 0.07). However, final body weight was similar between groups (*p* > 0.05). Body weights of male mink for the total experimental period are presented in Table 3.

### 3.2. Skins

Regarding grading of fur from live mink, neither female (*p* = 0.625) nor male (*p* = 0.535) animals presented statistically important differences between four groups. 

Nap size, skin size and final quality of skins were similar between the groups for both female and male mink (*p* > 0.05) (Table 4 and Table 5). Consequently, the prices of skins sold in the auction presented no differences between groups (*p* > 0.05).

### 3.3. Hair Re-Growth Assessment

**Figure 4:**
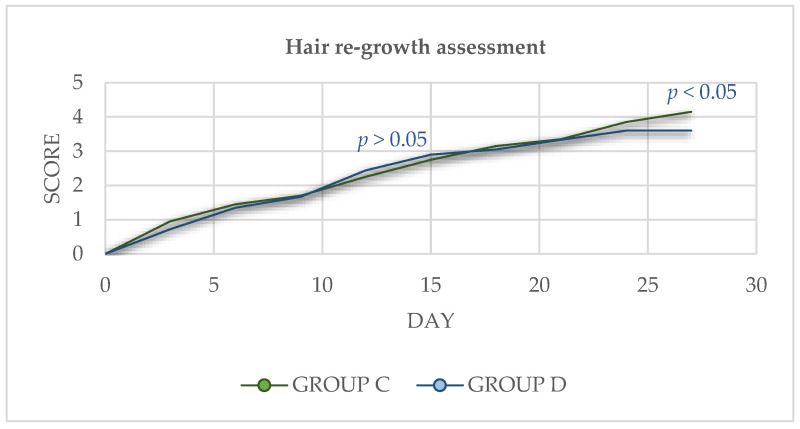
Hair re-growth assessment for the overall experimental period (27 days).

Spirulina supplementation did not affect the fur re-growth of mink during the overall trial period (*p* > 0.05). However, at the end of the experimental period, the Spirulina-treated group presented a better average scoring for hair re-growth (*p* < 0.05) (Figure 4). 

### 3.4. Histopathology, Immunohistochemistry and Morphometry

**Figure 5:**
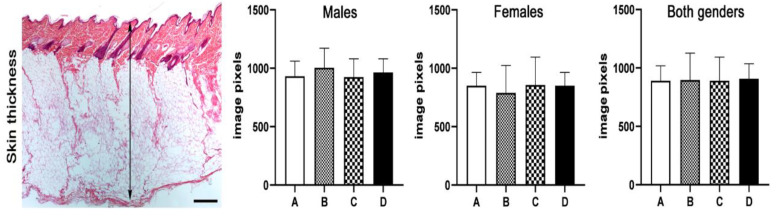
Skin thickness measurements (double-headed arrow) from female and male mink offspring. Experimental groups had comparable skin thickness at pelting period, irrespective of Spirulina diet status. Letters of x-axes correspond to the experimental groups (A, B, C, D).

Having found that feeding of mothers and/or their progeny with Spirulina did not affect fur quality and re-growth after shaving, we then morphometrically assessed skin thickness in the four groups of the study. Knowing that skin thickens during the anagen phase of hair follicle cycling and then is gradually reduced through the catagen phase to reach its thinnest size in the telogen phase, we next measured skin thickness by means of histomorphometry. We found that all four experimental groups had statistically comparable values for skin thickness, irrespective of spirulina feeding status. This outcome was similar in both male and female animals (Figure 5). These results match those of the hair shaving experiment, suggesting that dietary supplementation with spirulina does not work to accelerate hair follicle cycling.

**Figure 6:**
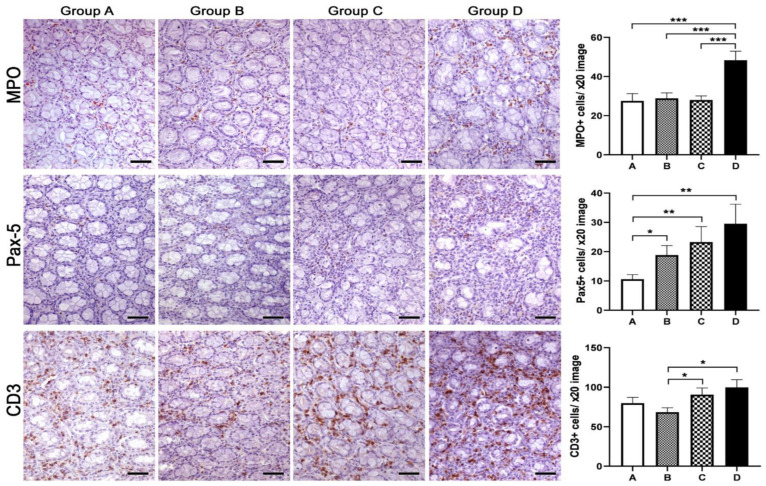
Effects of Spirulina treatments on inflammatory cells in the colonic mucosa of mink progeny. A Spirulina diet in progeny (group C), mothers (group B) or both (group A) worked to reduce the load of granulocytes (MPO), B-lymphocytes (PAX-5) and T-lymphocytes (CD3) in the colonic mucosa lamina propria in comparison to untreated controls (group D). Morphometric counts of immunohistochemically labeled inflammatory cells yielded statistically significant differences among experimental groups, as shown in the bar graphs. IHC: diaminobenzidine chromogen, hematoxylin counterstain. Scale bars: 50 μm. Numbers on the y-axes of bar graphs correspond to the means ± SEM of immune cell counts * *p* < 0.05, ** *p* < 0.01, *** *p* < 0.001. Letters of the x- axes correspond to the experimental groups (A, B, C, D).

Based on previous reports suggesting that Spirulina has immunomodulatory and anti-inflammatory properties, we next probed for the presence of granulocytes (MPO-positive), B-lymphocytes (PAX-5-positive) and T-lymphocytes (CD3-positive) in the colonic mucosa, MLN and spleen of mink using immunohistochemistry. Using quantitative histomorphometry, we compared the numbers of these key immune cells among the experimental groups of our study. In the colon (Figure 6), we found that group D, comprising mink that neither consumed Spirulina themselves nor had mothers who did, had statistically significantly more granulocytes in comparison to all three remaining groups, comprising mink that either consumed Spirulina themselves and/or had mothers who did (A vs. D, *p* = 0.001; B vs. D, *p* = 0.0009; C vs. D, *p* = 0.0004). A similar pattern was evident for B-lymphocytes, which were numerically greater in group D. In this case, however, group A, comprising progeny of mothers fed with Spirulina that also consumed Spirulina on a daily basis, stood out in terms of statistical significance when compared to all three remaining groups (A vs. B, *p* = 0.0043; A vs. C, *p* = 0.0411; A vs. D, *p* = 0.0043). Finally, colonic mucosa T-lymphocyte numbers were higher in groups C and D. In this case, however, a statistically significant difference was revealed only in the comparison with group B (B vs. C, *p* = 0.032; B vs. D, *p* = 0.012).

**Figure 7:**
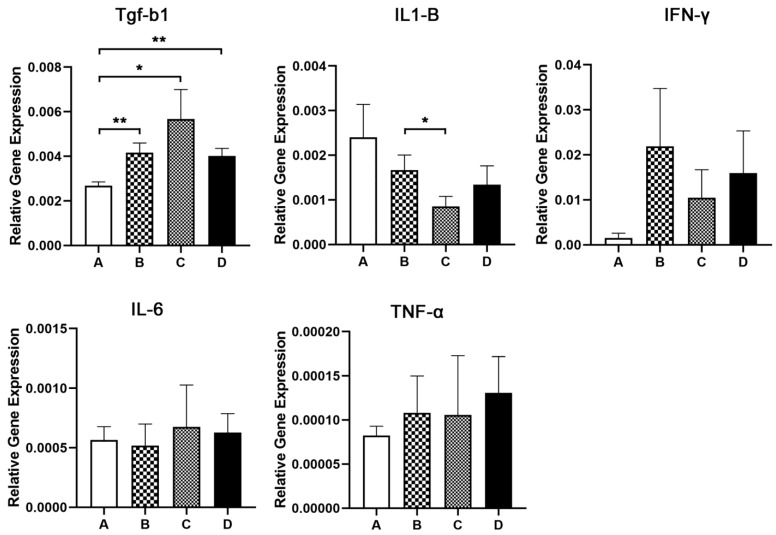
Expression of Tgf-β, IL1-β, IFN-γ, IL-6 and TNF-α in the colonic mucosa of the four experimental groups.* *p* < 0.05, ** *p* < 0.01. Letters of the x- axes correspond to the experimental groups (A, B, C, D).

To test whether these alterations in the immune cell component were connected to different cytokine mucosal milieus, we next quantitatively assessed the expression of Tgf-β, IL1-β, IFN-γ, IL-6 and TNF-α in the colonic mucosa. Statistically significant differences among groups were identified only for Tgf-β and IL1-β. Specifically, Tgf-β showed significantly lower levels in group A as compared to all three remaining groups (A vs. B, *p* = 0.0043; A vs. C, *p* = 0.0411; A vs. D, *p* = 0.0043), whereas IL1-β had significantly lower expression levels in group C in comparison to group B (*p* = 0.0411) (Figure 7). 

**Figure 8:**
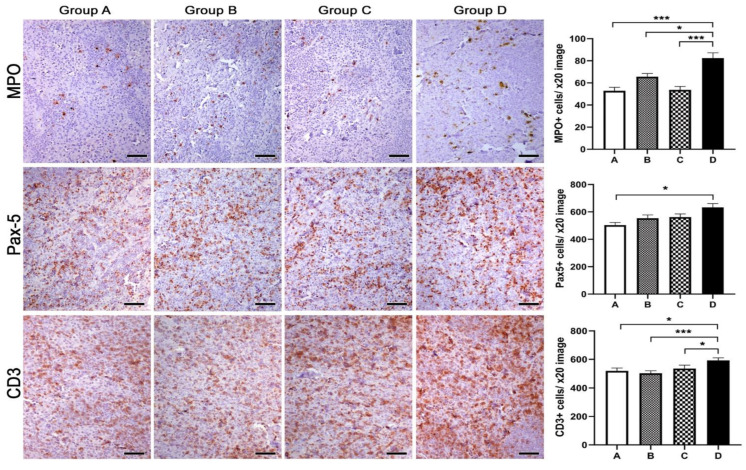
Effects of Spirulina treatments on inflammatory cells in paracortical areas of mesenteric lymph nodes of mink progeny. A Spirulina diet in progeny (group C), mothers (group B) or both (group A) connected with decreased numbers of granulocytes (MPO), B-lymphocytes (PAX-5) and T-lymphocytes (CD3) in the mesenteric lymph nodes compared to the non-treated control group (group D). Morphometric counts of immunohistochemically labeled inflammatory cells showed statistically significant differences among experimental groups, as highlighted in the bar graphs. IHC: diaminobenzidine chromogen, hematoxylin counterstain. Scale bars: 50 μm. Numbers on the y-axes of bar graphs correspond to the means ± SEM of immune cell counts * *p* < 0.05, *** *p* < 0.001. Letters of the x-axes correspond to the experimental groups (A, B, C, D).

In the MLN tissue, the prevalence of the control group D in terms of immune cell counts matched observations in the colon (Figure 8). Specifically, there were significantly more granulocytes in group D compared to groups A (*p* < 0.0001), B (*p* = 0.0138) and C (*p* < 0.0001). Group B also exhibited increased numbers of granulocytes compared to groups A (*p* = 0.0076) and C (*p* = 0.01). Likewise, B-lymphocytes were more abundant in group D; however, the difference only reached statistical significance in the comparison with group A (*p* = 0.0012). T-lymphocytes were also found in statistically significantly higher numbers in group D compared to all three remaining groups (A vs. D, *p* = 0.0174; B vs. D, *p* = 0.0009; C vs. D, *p* = 0.0471).

**Figure 9:**
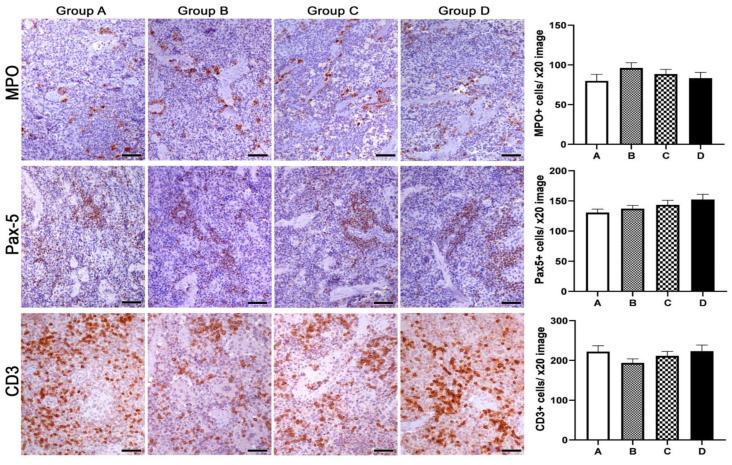
Effects of Spirulina treatments on inflammatory cells in the non-follicular areas of the spleen of mink progeny. Spirulina diet permutations involving progeny (group C), mothers (group B) or both (group A) did not alter the compositions of immune cell milieus compared to the non-treated control group (group D). Morphometric counts of immunohistochemically labeled inflammatory cells showed no statistically significant differences among experimental groups (bar graphs). IHC: diaminobenzidine chromogen, hematoxylin counterstain. Scale bars: 50 μm. Numbers on the y-axes of bar graphs correspond to the means ± SEM of immune cell counts. Letters of the x- axes correspond to the experimental groups (A, B, C, D).

Having found edible Spirulina to be connected to alterations in the immune cell component in local GI tract immune networks, we next examined the spleen to test for possible effects in systemic immunity. In contrast to what we found in the colonic mucosa and MLN, granulocyte and B- and T-lymphocyte numbers did not differ significantly in the spleen (Figure 9).

Taken together, our results suggested that, when introduced in the individual or maternal diet of mink, Spirulina had certain subclinical—yet palpable—effects in the GI tract immunity networks. This prompted us to examine whether Spirulina exerts this effect by altering the consistency of bacterial communities in the gut. To achieve this, we used fecal samples to analyze gut bacterial microbiome.

### 3.5. Gut Bacterial Microbiome

**Figure 10:**
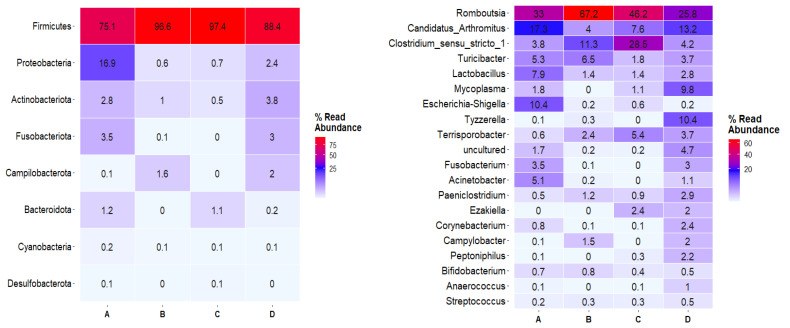
Heatmaps depicting the relative abundances of the major bacteria at the phylum (**left**) and genus (**right**) levels identified in each group. Numbers reflect the normalized relative abundance (%) within each group.

In total, 44,105,396 paired-end raw sequence reads were generated; after filtering, denoising and dereplication steps, the total number of reads was reduced to 37,366,008, with an average of 1,334,500 sequences per sample. These sequences were annotated to 2218 unique ASVs. *Firmicutes* was the most abundant phylum independently of the diet group, ranging from 75.1% in group A to 97.4% in group C, with *Clostridia* being the most abundant class, averaging 76.6% total relative abundance, followed by *Bacilli* (12.7%). Proteobacteria were the most abundant in group A (16.9%) compared to the other groups (0.6% in group B, 0.7% in group C, 2.4% in group D), with *Gammaproteobacteria* comprising 19.9% of the total relative abundance in mink samples. At the family level, *Peptostreptococcaceae* dominated the microbial communities in all groups (48.1% total relative abundance), followed by *Clostridiaceae* (22.5%), *Erysipelotrichaceae* (4.3%), *Lachnospiraceae* (3.45%) and *Lactobacillaceae* (3.4%). Differences in bacterial composition were observed at the genus level (Figure 10). In particular, *Romboutsia* was the most dominant genus in all groups, with higher relative abundance in groups B and C (67.2% and 46.3%, respectively). *Candidatus Arthromitus*, *Lactobacillus*, *Escherichia*-*Shigella*, *Acinetobacter* and *Bifidobacterium* were found in higher abundances in group A, while *Mycoplasma*, *Tyzzerela*, *Corynebacterium* and *Peptoniphilus* were mostly detected in group D. The relative abundance of *Clostridium sensu stricto 1* was increased in group B (11.3%) and C (28.5%) compared to group A (3.8%) and group D (4.2%). At the species taxonomic level, bacterial communities were comprised mostly of uncultured bacteria (>40% in groups A and D and approximately 20% in groups B and C). Several *Lactobacillus* species were identified: *Lactobacillus panis* was detected in group A at 5.5% relative abundance and at <0.3% in the other three groups, *Lactobacillus reuteri* was detected only in group A, while *Lactobacillus sakei* was detected at a higher percentage in group D (1.5% compared to <0.4% in the rest of the groups).

**Figure 11:**
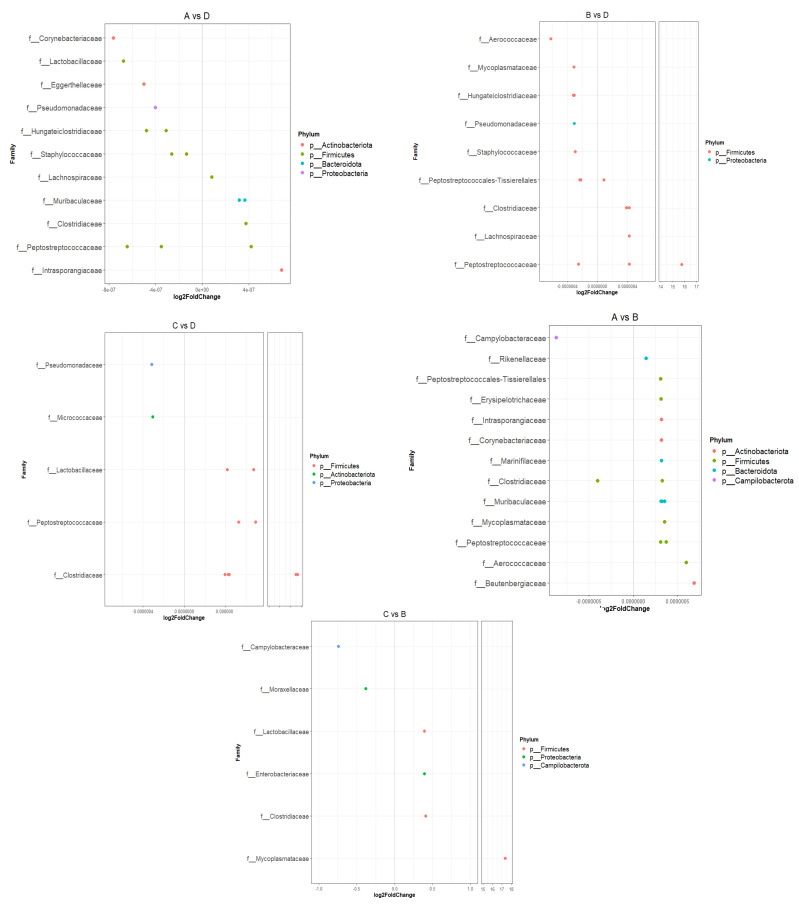
Differentially abundant ASVs depicting the log2fold differential abundance of the different families for pairwise comparisons among groups (DESeq2 analysis). Differences were considered significant with an adjusted *p*-value of 0.01. Log2fold values greater than zero indicate an increase (enrichment) in the relevant ASVs of the test group (A, B or C), whereas negative values indicate higher abundances in the control group (D).

The impact of different dietary supplementation was further investigated by testing for differential abundances among test (A and C) and control (B and D) groups (Figure 11). DESeq2 pairwise comparisons of groups A and C versus the control group B revealed 17 and 6 differentially abundant ASVs, respectively, with adjusted *p* value < 0.01. In both cases, the *Campylobacteraceae* family was enriched in group B. In pairwise comparison of group A against B, ASVs belonging to *Erysipelotrichaceae*, *Corynebacteriaceae*, *Muribaculaceae*, *Mycoplasmataceae* and *Aerococcaceae* were significantly enriched in group A, while pairwise comparison of group C versus B revealed that *Lactobacillaceae*, *Enterobacteriaceae*, *Clostridiaceae* and *Mycoplasmataceae* ASVs were enriched in group C. Pairwise comparisons of groups A and C versus the control group D resulted in 16 differentially abundant ASVs in group A versus D, while 11 ASVs were found to be differentially abundant between groups C and D. In all cases ASVs, belonging to the *Pseudomonadaceae* family were enriched in samples of group D, while members of the *Peptostreptococcaceae* family were significantly enriched in groups A and C. In addition, group C showed significantly higher enrichment of ASVs classified in the *Lachnospiraceae* and *Clostridiaceae* families compared to group D. Pairwise comparison of group A versus group D revealed that ASVs classified in the *Intrasporangiaceae*, *Clostridiaceae*, *Muribaculaceae* and *Lachnospiraceae* families were all enriched in group A. Finally, pairwise comparison of control groups (B and D) revealed that group B had significantly higher abundances in ASVs belonging to *Clostridiaceae* and *Lachnospiraceae* families compared to group D. 

## 4. Discussion

In the present study, the effects of dietary supplementation with *Spirulina platensis* on the growth, skin and pelt quality, immune system and bacterial gut microbiome of mink were investigated. 

In general, the effects of Spirulina supplementation on animal performance and growth rate have been extensively studied [39]. Although many variations were observed regarding body weight and body weight gain across the whole experimental period, the final body weights for female and male animals did not present any difference between the groups. However, it should be noted that male mink during the second month of the experiment (August) presented the highest body weight gain compared to the other groups. In light of the fact that feed intake may be suppressed during increased environmental temperatures, it can be hypothesized that feed efficiency may have been improved in the group supplemented with Spirulina. However, due to practical reasons, it was not possible to accurately measure the feed intake of all animals. Mirzaie et al. reported similar results for broiler chickens that were exposed to high ambient temperature; broilers fed with Spirulina presented a numerically improved feed conversion ratio [40]. Our results are in accordance with those that we have previously published, which showed that Spirulina supplementation did not affect the final body weight of either mink dams during their reproduction period or of their kits’ final body weight at weaning [41]. Similar results have also been presented for broiler chickens [39,42] and growing rabbits [43]. In contrast, other authors have reported that Spirulina supplementation resulted in improved body weight in broilers [44], growing rabbits [45] and weaned piglets [46]. These controversial results may indicate that the impact of dietary inclusion of Spirulina on animal growth depends on many factors, including dose level, and further research is needed. 

Spirulina is a cyanobacterium that is widely used in human and animal consumption due to its properties [47]. Among the most interesting findings is that Spirulina demonstrates antimicrobial activity and, consequently, microbial-modulating properties [48,49]. To investigate the effects of dietary inclusion of Spirulina on bacterial communities of mink gut, we proceeded with stool bacterial flora microbiome analysis. According to previous reports on various animal species, alterations in gut microbial communities after systematic treatment with Spirulina can be observed [4,27,48,50]. 

In rats, Spirulina decreased the abundance of *Proteobacteria* and the *Firmicutes/Bacteroidetes* ratio in fecal samples when they were supplemented with a high- fat diet [51]. However, this was not the case in our study, since the phylum *Firmicutes* was found in great relative abundances in all groups (over 75.1%). This result is in line with previous reports that have indicated that the gut bacterial microbiome of mink is mainly dominated by the phyla *Firmicutes*, *Proteobacteria* and *Fusobacteria* [3,17]. Our results also revealed that *Clostridia* were the most abundant class, independently of the diet group, which agrees with other studies on mink. In fact, *Clostridia* were found to dominate bacterial communities in farmed mink populations fed with a regular diet and after three-day fasting [15,52]. *Clostridia* species have been previously described to be associated with abortion, metritis and mortality rate in mink [53,54]. However, *C. limosum*, which is considered the main bacterium responsible for these diseases, was absent from our dataset.

Growth of lactic acid bacteria (LAB) is promoted by Spirulina, which presents both probiotic and prebiotic effects while, at the same time, inhibiting the increase in harmful microbes in the gut [49,51,55]. In addition, recent studies have revealed that Spirulina benefits the growth of *Akkermansia*, *Lactobacillus* and *Butyricimonas* and simultaneously suppresses the growth of *Clostridium* and *Dorea* [56,57,58]. The increase in LAB with a diet supplemented with Spirulina was also observed in our experiment: *Lactobacillus* was most abundant in group A compared to the other diet groups. In addition, many other LAB species were detected in our study but at low relative abundances. In particular, *Lactobacillus panis* was detected in higher abundance in group A compared to the other three groups. The same pattern was observed for *Lactobacillus reuteri*, which was detected only in group A, while *Lactobacillus sakei* was detected at a higher percentage in group D. However, supplementation with spirulina did not impact the growth of *Escherichia/Shigella* pathogens since they were detected in group A in higher relative abundance compared to the other groups, in which they were detected in traces (below 0.6%). 

At the genus level, *Romboutsia* was the most dominant bacterium in all groups, with higher relative abundances in groups B and C. While previous studies in rats revealed that spirulina decreased the abundance of *Romboutsia* in gut microbiome [57], this was not the case in our experiment. *Romboutsia* members are mainly gut inhabitants and they contribute to aspects of metabolism, such as carbohydrate utilization, fermentation of single amino acids, anaerobic respiration and metabolic end products [59]. Although this genus was also identified as dominant in mink gastrointestinal tracts at 116 and 205 days after birth (almost the same time period with which the time-point fecal sampling was conducted in our experiment), its exact role in the mink gut microbiome remains to be explored [52].

Since the bacterial communities and abundances within the gastrointestinal tract were not severely impacted by the addition of Spirulina in mink diet, we subsequently tested for certain taxa that were significantly overrepresented in certain groups through DA analysis. DA analysis revealed that the *Campylobacteraceae* family—and, in particular, *C. jejuni*—was enriched in group *B. Campylobacter* is a bacterial pathogen that has been reported as a cause of foodborne illness and can result in acute gastroenteritis [60]. The enrichment of this species in the control group implied that addition of Spirulina may have inhibited bacterial growth in mink fed with *Spirulina platensis*. In addition, comparison of animals that received spirulina to the control group D revealed that members of the *Pseudomonadaceae* family were more abundant in group D, also implying a protective role for spirulina in the gastrointestinal tract of mink. This finding is of high importance since *Pseudomonas* aeruginosa can cause hemorrhagic pneumonia, an infectious disease, in mink, threatening the mink industry [61]. Moreover, DA analysis revealed that members of the *Peptostreptococcaceae* family, with *Romboutsia* being the main representative, were significantly enriched in groups A and C compared to control animals (group D). Members of this family were also identified as the most abundant species in the colon of mink fed with the seaweeds *Saccharina latissima* and *Palmaria palmata* [62]. The question of whether the gut microbiome in mink can be manipulated with the addition of seaweed in the diet remains to be explored, since previous authors found small effects on the bacterial communities associated with the intestinal mucosa in mink. This finding was similar to our results; although differences were observed in gut bacterial communities, further research is required to support the notion that Spirulina benefits mink health and pelt quality. Due to the fast transit of food within the gastrointestinal tract of mink, bacterial populations might not have enough time to populate and grow in the gastrointestinal tract, although Williams et al. pointed out that quantitative and qualitative characteristics of dietary inputs can influence the bacterial populations present in the lumen of the gastrointestinal tract in mink and those associated with the mucosa [16]. To assess the worth of Spirulina in the gut microbiome in mink, other factors should be co-evaluated, including genetics and the immune status of the host. 

The constant and tight interaction between the gastrointestinal tract and the immune system has been extensively described in many published reports [5,63,64]. Inflammatory responses in the gut may be a result of a disruption in the interaction between the intestinal microbiome and regional immune defense system [48]. Elsewhere, it has been suggested that alterations in microbiota may result in immune-mediated diseases, as microbial communities affect both barrier surfaces and remote organs [64,65,66]. For this reason, we undertook an immunohistochemical analysis of immune cells in the colonic mucosa, mesenteric lymph nodes and spleen of mink. 

According to previous studies, Spirulina and/or extracts of Spirulina demonstrate anti-inflammatory activity in enhancing mucosal immunity, production of antibodies and activation of T-cells [67]. Spirulina preparations participate in both the metabolic activity of neutrophils and the inactivation of superoxide radicals produced by functional neutrophils [68]. Elsewhere, it has been found that Spirulina increases the phagocytic activity of macrophages, causes natural killer cells to accumulate in tissues and mobilizes T and B cells [69]. Our findings suggest that dietary supplementation with Spirulina in mothers and/or offspring has a long-term effect on the number of inflammatory cells in the regional gut immune system. This subclinical effect is mainly represented by the significant reduction in the number of neutrophils, and it is indicative of a beneficial reduction in the immune tone in the colonic mucosa and mesenteric lymph nodes. However, this long-term effect does not extend systematically, as it is not also reflected in the spleen. 

To examine further the effect of Spirulina supplementation on the immune system of mink, we undertook quantitative assessment of pro-inflammatory cytokine expression in the gut. Cytokines are secreted by numerous types of cells in the gut and they variously participate in local immune responses [70]. According to previous studies, Spirulina supplementation inhibits overproduction of pro-inflammatory cytokines in gut mucosa [23,28,51,71]. Our results suggested that the quantitative analysis of cytokine gene expression in colonic mucosa confirmed the finding of the immunohistochemical analysis that spirulina affects gastrointestinal immune cells. These findings, although limited, are remarkable, as they were obtained in intestinal mucosa of healthy animals with no clinical signs of enteritis. Tgf-β is a pleiotropic cytokine with an important role in suppressing cell proliferation. The low levels in group A were probably related to the better homeostatic balance between proliferation and cell death in the colonic epithelium. It could also be hypothesized that these effects, especially in group A, could also be related to the changes in gut bacteria communities. It is interesting to note that, in group A, the LAB population in the growing mink was increased. LAB are known to be related to improved intestinal health [72]. Therefore, the favorable immune profile in growing minks with mothers that were supplemented with Spirulina during the reproductive period could have also been related to the modulation of gut flora in the reproductive mink. Such a maternal effect could not be proven directly in our study, as no dams’ gut microbiome profile was analyzed. In piglets, it has recently been shown that maternal treatment with probiotics or synbiotics improved immune response by altering gut microflora [73]. It is plausible that a similar mechanism may have occurred in our case. The results showing that IL1-B presented lower levels in group C compared to group B need further investigation as they suggest, in addition to the direct dietary effect of Spirulina, the presence of a complex immune-tolerance phenomena in relation to whether or not maternal supplementation with dietary Spirulina has taken place.

Mink are bred worldwide for their fur. Fur quality and pelt size are the main traits that configure the final price of the skin [74,75]. Elsewhere, it has been found that dietary protein levels affect hair growth and hair properties in mink pelts [76], while probiotic supplementation improves pelt quality [77]. According to previous studies, supplementation with algae may result in healthy skin and a lustrous coat [78]. Similar results have also been demonstrated for probiotics [12,14]. The gut–brain–skin axis has been previously described, and it has been indicated that gut microbial communities can interact with remote organs, such as skin [6,79]. Although Spirulina’s effect on skin has been extensively studied in terms of in vitro and in vivo external use with great results [29,80,81,82], to the best of our knowledge, there is no published study reporting an oral/systematic treatment with it. 

To examine whether dietary inclusion of Spirulina could affect the final quality of mink skin, we undertook qualitative and quantitative analyses of skins. Hair re-growth assessment of the shaved area in mink did not present any difference between the groups according to the grading of fur quality evaluators. Furthermore, skin thickness was similar among the experimental groups, suggesting that Spirulina supplementation at that dose level presented no effect on either quality or on the histomorphometrical view of mink skins. 

Overall, according to our and other previously published results, it can be suggested that dietary inclusion of Spirulina in mink may lead to improved overall health status without affecting the quality of their fur. However, further research is needed.

## 5. Conclusions

The results of the present study indicate that Spirulina decreased the markers of subclinical inflammation of the mink gastrointestinal system. Moreover, differences in the bacterial communities’ composition among groups were observed. Dietary inclusion of Spirulina at a dose of 100 mg/kg of body weight did not affect skin and pelt quality. However, further investigation is required to determine the exact mechanism by which Spirulina participate in the gut–immune system axis, as well as the maternal effect of the supplement.

## Figures and Tables

**Table 1:** Primers used for gene expression analysis.

Primer	Sequence (5′-3′)	Positions ^1^	Amplicon Size (bp)	Concentration (nM)	Annealing Temperature (°C)
Tgf-b1-F	GCTAGACCGCTTGCTTCAGG	262–281	111	100	61
Tgf-b1-R	TGGTCCTCCTTCTGTTCCCC	353–372			
Il-1b-F	CCTGAACCCACCAGTGAAGT	110–129	144	250	62
Il-1b-R	TTGAAGCTGGATGCCCTCCT	234–253			
Ifng-F	AGATGGTGGGCCTCTTTTCT	138–157	155	200	61
Ifng-R	CCTTGATGGTATCCATGCTCCT	271–292			
Il6-F	ACCTAGAGGCCAACTATGAGGG	383–404	128	100	61
Il6-R	ATCTGTGGTAGGGTTGGGGG	491–510			
Tnf-a-F	CCAGATGGCCTCCAACTAATCA	11–32	110	400	61
Tnf-a-R	CCCTCAGCTTCAGGGTTTGC	101–120			
Gapdh-F	GGGTGTGAACCACGAGAAGT	6–25	122	250	61
Gapdh-R	GTCCCTCCACAATGCCGAAG	108–127			

^1^ According to gene sequences with the following accession numbers in the Ensembl or GenBank databases: Ensembl-Tgf-b: ENSNVIT00000018937.1, GenBank-Il-1b: HAAF01000134.1, Ifng: KJ888148.1, Il6: EU753358.1, Tnf-a: GU327784.1, Gapdh: KM025344.1.

**Table 2:** Descriptive statistics for body weights of female mink (mean and standard deviation) for the whole experimental period (*n* = 25 per group).

	Group				
Grams	A (±SD ^1^)	Β (±SD ^1^)	C (±SD ^1^)	D (±SD ^1^)	*p*-Value
BW1	949.28 (112.39)	898.80 (85.45)	931.84 (84.99)	882.44 (100.02)	0.065
BW2	1429.72 (149.40)	1361.08 (139.95)	1408.08 (152.69)	1402.48 (150.65)	0.077
BW3	4699.36 (200.22)	1634.00 (188.66)	1724.24 (177.04)	1671.48 (231.16)	0.345
BW4	1913.36 (246.11)	1833.76 (210.72)	1949.04 (190.19)	1883.76 (284.79)	0.383
BW5	2008.00 (297.04)	1934.16 (248.89)	2044.32 (230.24)	2027.92 (307.83)	0.151

^1^ SD: standard deviation.

**Table 3:** Descriptive statistics for body weights of male mink (mean and standard deviation) for the whole experimental period (*n* = 25 per group).

	Group				
	A (±SD ^1^)	Β (±SD ^1^)	C (±SD ^1^)	D (±SD ^1^)	*p*-Value
BW1	1175.68 (168.14)	1176.32 (297.04)	1224.16 (156.69)	1116.36 (121.85)	0.077
BW2	2064.16 (183.34)	2042.6 (123.53)	2089.08 (239.71)	1966.88 (187.06)	0.749
BW3	2826.76 (250.37)	2694.24 (168.36)	2802.32 (304.56)	2643.08 (282.42)	0.127
BW4	3498.32 (357.05)	3316.68 (333.23)	3397.04 (376.61)	3246.36 (392.64)	0.133
BW5	3945.84 (458.92)	3735.28 (432.22)	3782.44 (499.67)	3671.84 (478.51)	0.197

^1^ SD: standard deviation.

**Table 4:** Descriptive statistics (mean and standard deviation) for skins of female mink (*n* = 6 per group).

	Group				
Trait	A (±SD ^1^)	B (±SD ^1^)	C (±SD ^1^)	D (±SD ^1^)	*p*-Value
Skin size	2.00 ± 0.89	1.67 ± 1.21	1.83 ± 1.17	1.50 ± 0.84	0.618
Nap size	1.83 ± 0.41	1.83 ± 0.41	1.50 ± 0.55	1.50 ± 0.55	0. 392
Quality	1.83 ± 0.75	1.66 ± 0.82	2.00 ± 0.63	1.83 ± 0.75	0.948
Grading	4.33 ± 0.52	4.17 ± 0.75	3.83 ± 0.75	4.33 ± 0.52	0.625
Price	25.83 ± 5.27	32.17 ± 5.23	28.83 ± 5.19	28.00 ± 6.20	0.279

^1^ SD = standard deviation.

**Table 5:** Descriptive statistics (mean and standard deviation) for skins of male mink (*n* = 6 per group).

	Group				
Trait	A (±SD ^1^)	B (±SD ^1^)	C (±SD ^1^)	D (±SD ^1^)	*p*-Value
Skin size	2.83 ± 0.75	2.83 ± 0.41	2.5 ± 0.55	2.5 ± 0.83	0.504
Nap size	1.50 ± 0.55	1.33 ± 0.52	1.83 ± 0.41	1.5 ± 0.55	0.363
Quality	1.66 ± 0.52	2.83 ± 0.98	2.00 ± 0.89	2.17 ± 1.17	0.469
Grading	4.33 ± 0.82	4.33 ± 0.52	4.00 ± 0.89	4.5 ± 0.55	0.535
Price	50.17 ± 10.55	51.50 ± 3.08	51.83 ± 2.04	50.5 ± 6.63	0.965

^1^ SD = standard deviation.

## Data Availability

The data presented in this study are available on request from the corresponding author.
